# 

**Published:** 1893-11-18

**Authors:** 


CONTENTS.

**A Learned Profession** - - - - - - - - - - - 97
**Around the Hospitals** - - - - - - - - - - - 98
**Therapeutics in Diphtheria.** By Sir B. W. Richardson, M.D., F.R.S. - - - - - - - - - - - - 99
**Modern Biology.** III.—On the Minute Structure of Cells - - 100
**The Study of Man.** I.—Object and Scope - - - - - - 101
**The Field of Science** - - - - - - - - - - 102
**Annotations.**—Malthus Redivivus—A B C Diet—Progress in Port Sanitation - - - - - - - - - - - - - 103
**Notes and News** - - - - - - - - - - - - 104
**Scraps and Gleanings** - - - - - - - - - - 112
**The Book World of Medicine and Science** - - - - vii
**The Hospital Clinic—**
London Hospital—The Treatment of Pneumonia - - - - 105
Royal Infirmary, Edinburgh—The Treatment of Erysipelas 107
New Drugs and Preparations—Uric Acid Solvents in Gout and Gravel - - - - - - - - - - - - - 108
New Appliances and Things Medical—The Improved Clinical Chart Block and Case - - - - - - - - - - 108
**The Institutional Workshop—**
Within the Hospitals—Middlesex Hospital - - - - 109
Hospital Construction—The London Hospital New Buildings - 110
Hospital Meetings—St. George's Hospital - - - - - 111
Practical Departments—Glass-topped Locker - - - - 112

EXTRA SUPPLEMENT.—THE NURSING MIRROR.

**News from the Nursing World.**—Our Christmas Competition — A Searching Investigation Imperative! — Special Training—Wanted, Trained Nurses—"Boys will be Boys"—Prudent Precautions—An Honoured Guest—Nursing Sisters in India—Grateful Acknowledgment—Unique Testimonials - lxi

**On the Nursing of Diseases of the Stomach.** II.—Vomiting - - - - - - - - - - - - - - lxiii

**Death in Our Ranks** - - - - - - - - - - lxiii

**Women's Work.**—Female Medical Aid for the Women of India. By Mrs. Scharlieb, M.D. I.—The Women of India - - lxiv

**Nursing in the United States.**—Proper Organisation of Training Schools (*continued*). By Miss Darche - - - lxiv

**Notes and Queries** - - - - - - - - - - - lxv

**Nursing in Switzerland.** By Dr. Charles Kraft - - lxv

**Presentation** - - - - - - - - - - - - - lxvi

**Prince Alfred Hospital, Sydney.**—The Nurses' Dining-room - - - - - - - - - - - - - - lxvi

**Wants and Workers** - - - - - - - - - - - lxvi

**Our American Letter** - - - - - - - - - - lxvii

**Everybody's Opinion.**—The New Order of Practitioners: Ask the Doctor - - - - - - - - - - - - - lxvii

**The Muses Looking Glass.**—A Hospital "Sing" - - lxviii

**Where to Go.**—Pictures - - - - - - - - - lxviii

**The Book World for Women and Nurses** - - - - lxix

